# Supplementary material for: Structural and biophysical correlation of anti-NANP antibodies with in vivo protection against P. falciparum
Source: Nat Commun. 2021 Feb 16;12:1063. doi: 10.1038/s41467-021-21221-4 (PMC7887213; doi:10.1038/s41467-021-21221-4)
Supplement: Supplementary file 1 — Supplementary information [file 41467_2021_21221_MOESM1_ESM.pdf]

Supplemental Information for

Structural and biophysical correlation of anti-NANP antibodies with *in vivo* protection against *P. falciparum*

Tossapol Pholcharee[1], David Oyen[1†], Yevel Flores-Garcia[2], Gonzalo Gonzalez-Paez[1], Zhen Han[1‡], Katherine L. Williams[3], Daniel Emerling[3], Wayne Volkmuth[3], Emily Locke[4], C. Richter King[4], Fidel Zavala[2], Ian A. Wilson[1,5*]

[1]Department of Integrative Structural and Computational Biology, The Scripps Research Institute, La Jolla, CA 92037, USA.
[2]Malaria Research Institute, Johns Hopkins Bloomberg School of Public Health, Baltimore, MD 21204, USA.
[3]Atreca Inc., South San Francisco, CA 94080, USA.
[4]PATH's Malaria Vaccine Initiative, Washington, DC 20001, USA.
[5]The Skaggs Institute for Chemical Biology, The Scripps Research Institute, La Jolla, CA 92037, USA.

*Corresponding author: Email: wilson@scripps.edu (I.A.W.)
†Current address: Pfizer Inc., San Diego, CA 92121, USA.
‡Current address: Wondfo USA Co., Ltd., San Diego, CA 92121, USA.

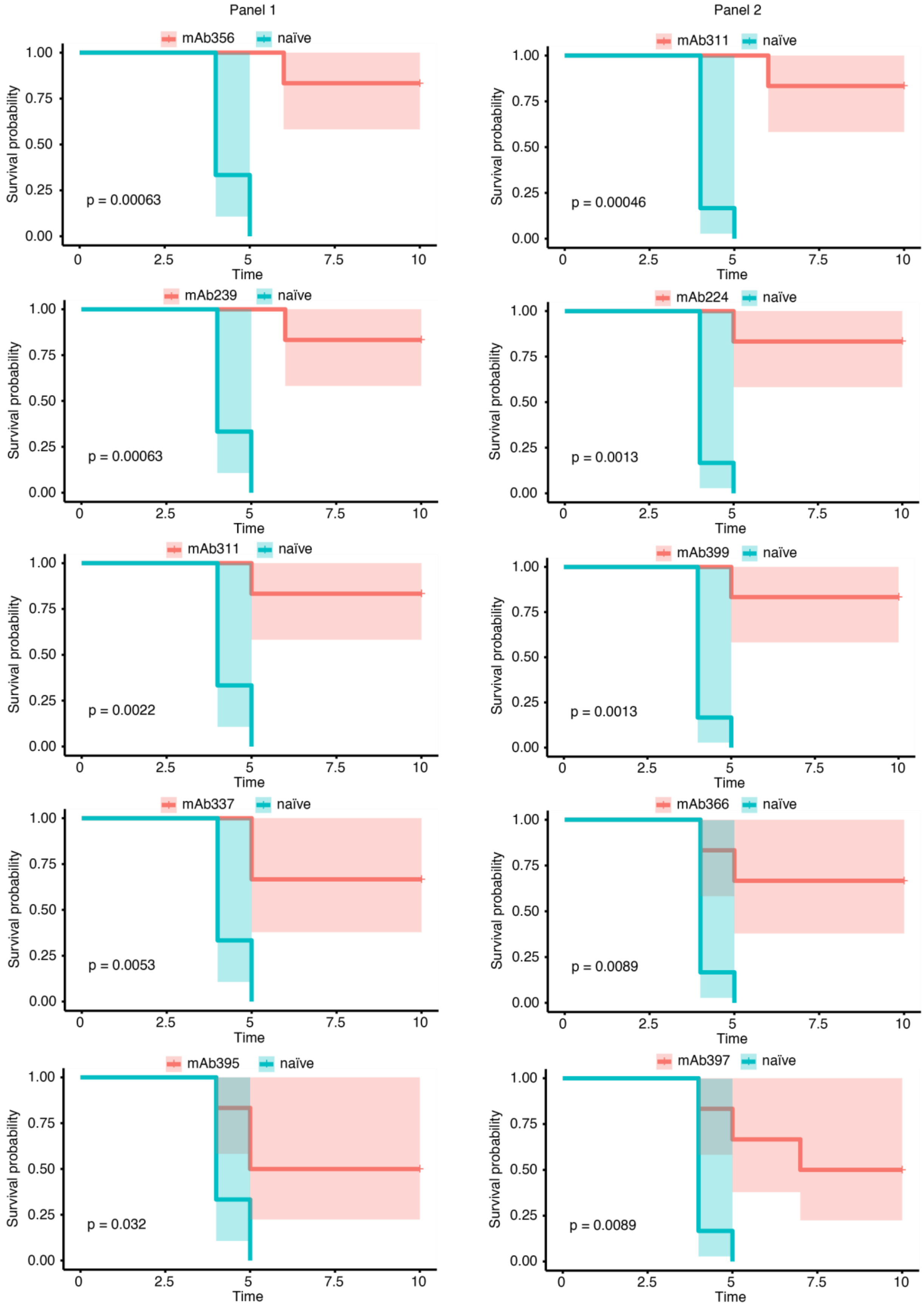

Panel 1
Panel 2
mAb356
naïve
p = 0.00063
mAb311
naïve
p = 0.00046
mAb239
naïve
p = 0.00063
mAb224
naïve
p = 0.0013
mAb311
naïve
p = 0.0022
mAb399
naïve
p = 0.0013
mAb337
naïve
p = 0.0053
mAb366
naïve
p = 0.0089
mAb395
naïve
p = 0.032
mAb397
naïve
p = 0.0089
Survival probability
1.00
0.75
0.50
0.25
0.00
Time
0
2.5
5
7.5
10

Supplementary Fig. 1. Individual survival curves from the parasitemia assay with 300 µg antibody. Parasite-free mice were passively immunized with 300 µg of the indicated antibody before challenge with bites from infected mosquitoes and compared to naïve control mice. A log rank test was used, and the p-value is shown. The shaded area indicates the 95% confidence interval (*N* = *6*). Source data are provided as a Source Data file.

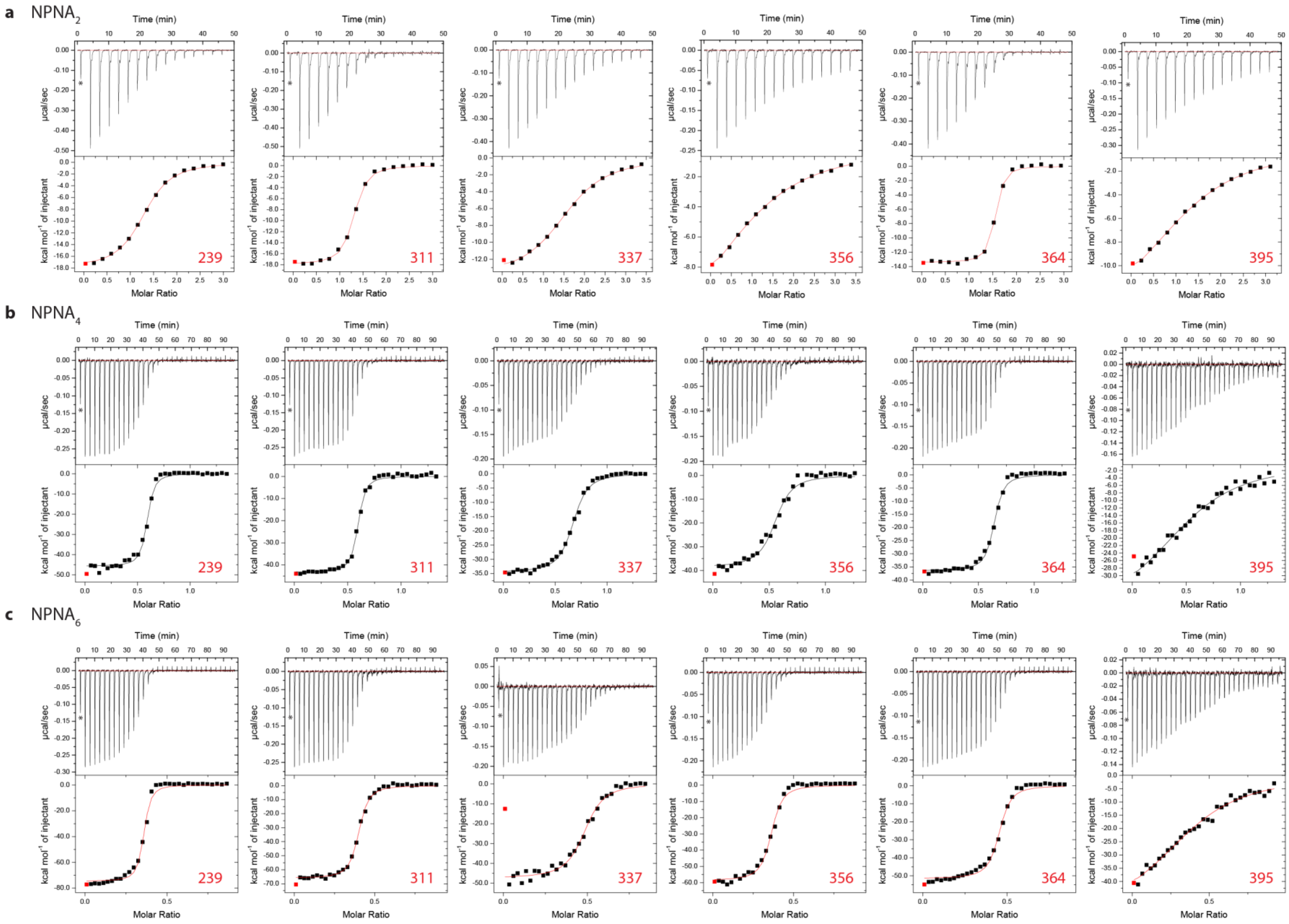

a NPNA2
b NPNA4
c NPNA6
Time (min)
µcal/sec
kcal mol-1 of injectant
Molar Ratio
239
311
337
356
364
395

Supplementary Fig. 2. ITC binding curves for *IGHV3-33* Fabs. ITC binding data are shown for each indicated Fab with: **a** 8-mer peptide Ac-NPNANPNA-NH_2, **b** 16-mer peptide Ac-NPNANPNA NPNANPNA-NH_2, and **c** 24-mer Ac-NPNANPNA NPNANPNA NPNANPNA-NH_2. The first data point is not included in the fit as is standard practice and indicated by an asterisk and red square. Source data are provided as a Source Data file.

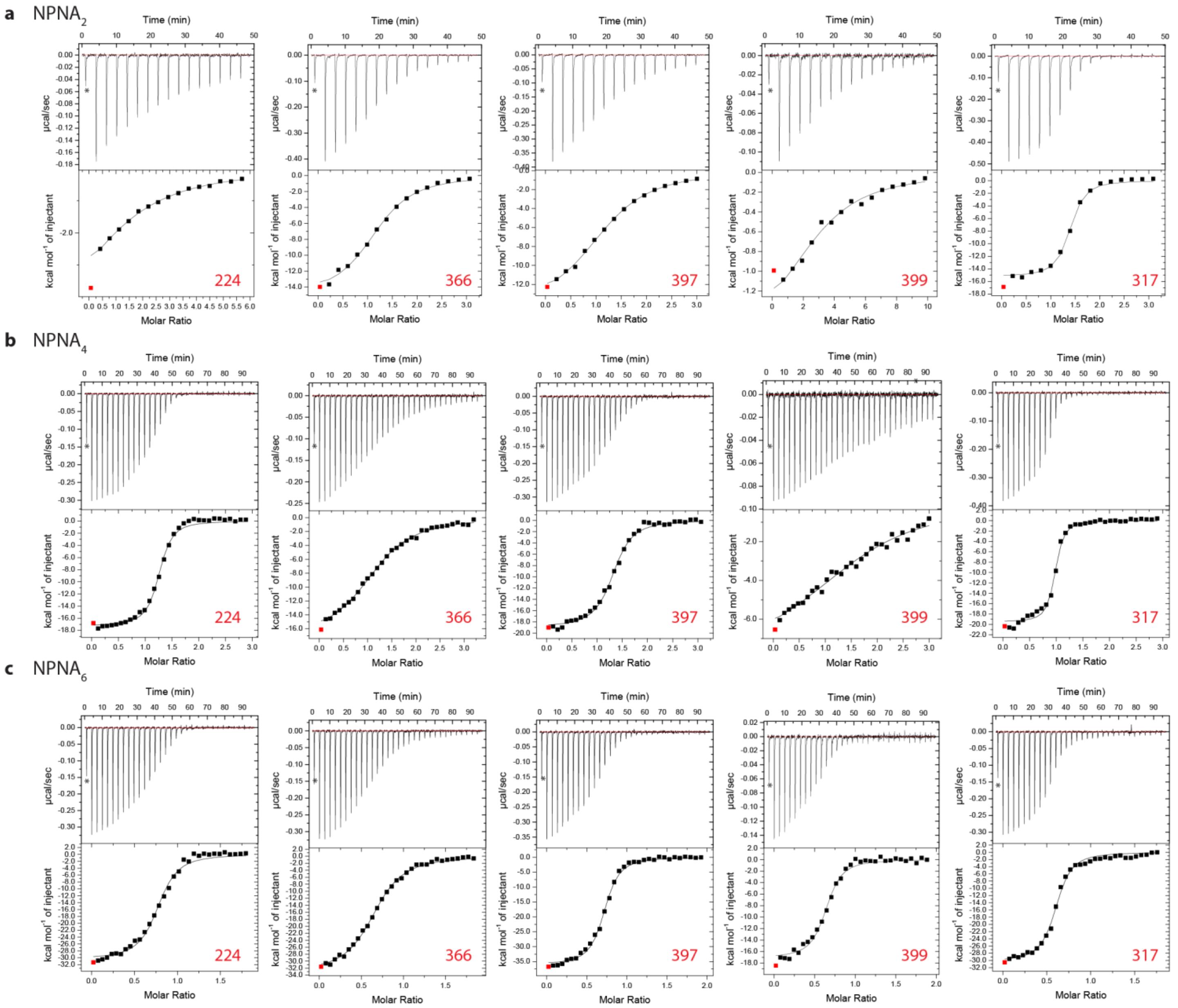
a NPNA2
b NPNA4
c NPNA6
Time (min)
µcal/sec
kcal mol-1 of injectant
Molar Ratio
224
366
397
399
317

Supplementary Fig. 3. ITC binding curves for Fab 224, 366, 397, 399, and 317. ITC binding data are shown for each indicated Fab with: **a** 8-mer peptide Ac-NPNANPNA-NH_2, **b** 16-mer peptide Ac-NPNANPNA NPNANPNA-NH_2, and **c** 24-mer Ac-NPNANPNA NPNANPNA NPNANPNA-NH_2. The first data point is not included in the fit as is standard practice and indicated by an asterisk and red square. Source data are provided as a Source Data file.

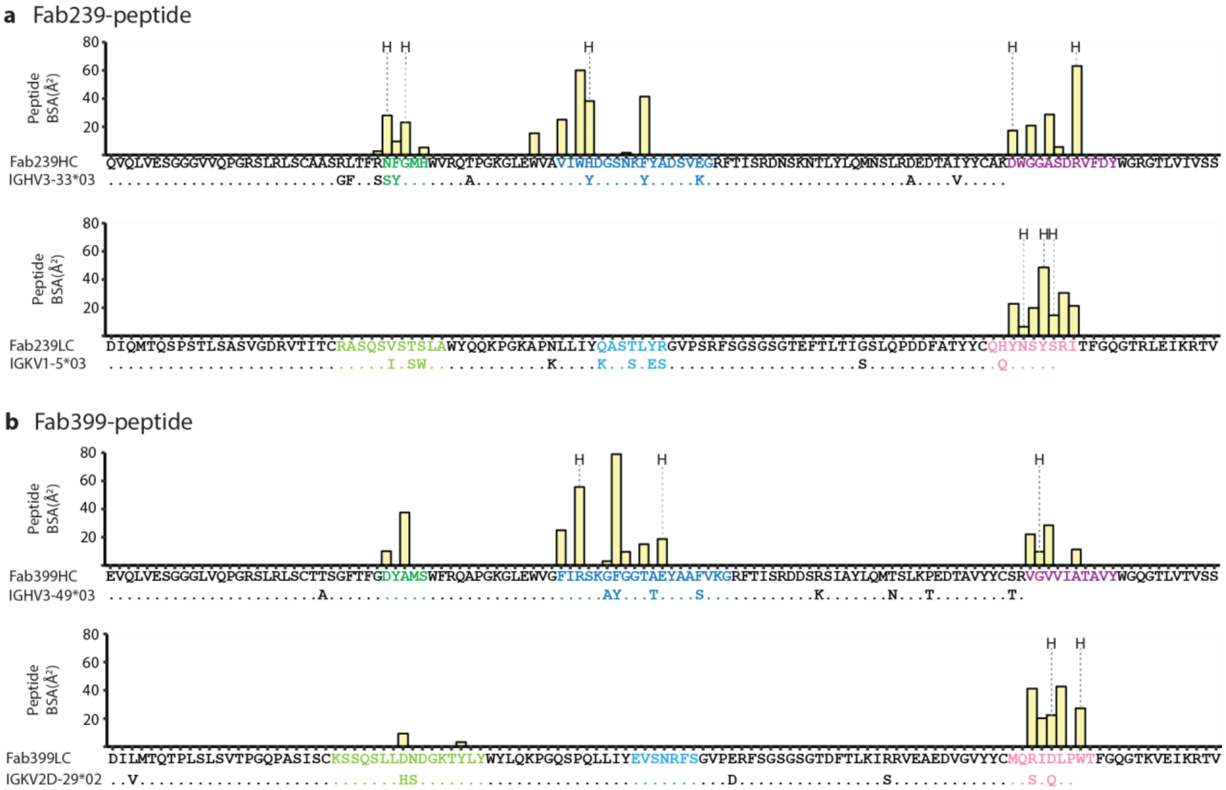

Supplementary Fig. 4. Individual residue contributions to the BSA of the Fab-peptide interface. The buried surface areas (BSAs) are shown in yellow bars for the heavy and light chains of **a** Fab239 and **b** Fab399. CDRs are colored as green, blue, magenta, light green, light blue, and pink for CDR H1, H2, H3, L1, L2, and L3, respectively. Additionally, the alignment between the Fab heavy/light chain sequences and germline *IGHV* and *IGKV* gene sequences also indicates which residues are somatically mutated. The letter “H” marks residues that are engaged in hydrogen bonds.

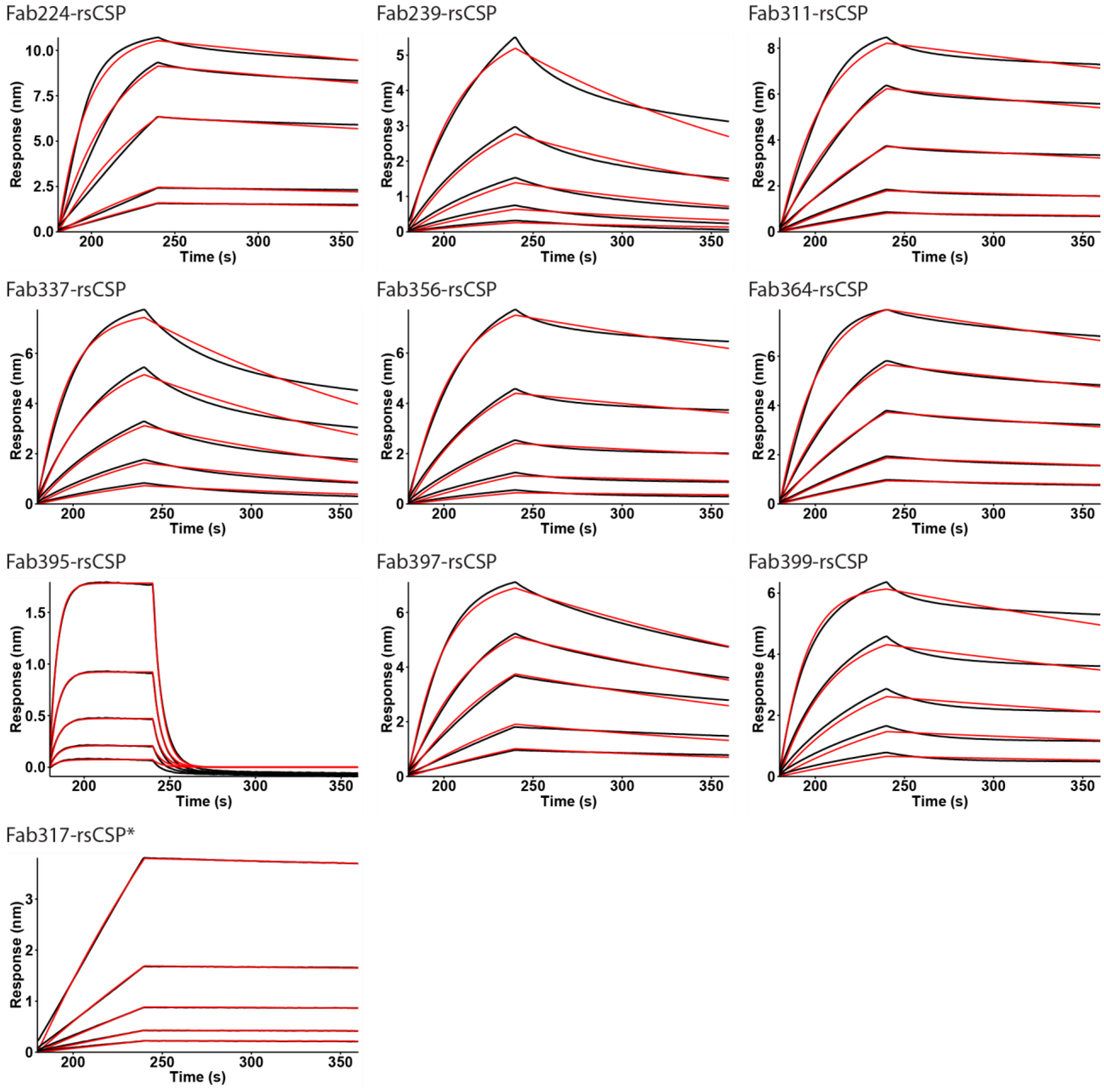

Supplementary Fig. 5. Kinetics of binding for mAbs analyzed in this study with rsCSP. Binding was assessed using bio-layer interferometry (BLI). Binding curves are shown in black and fitted curves (1:1 binding model) are shown in red. From the top to bottom curves, Fab concentrations are 1000, 500, 250, 125, and 62.5 nM, respectively (except for Fab317 where serial dilutions are 250, 125, 62.5, 31.25, 15.63 nM, respectively). Source data are provided as a Source Data file.

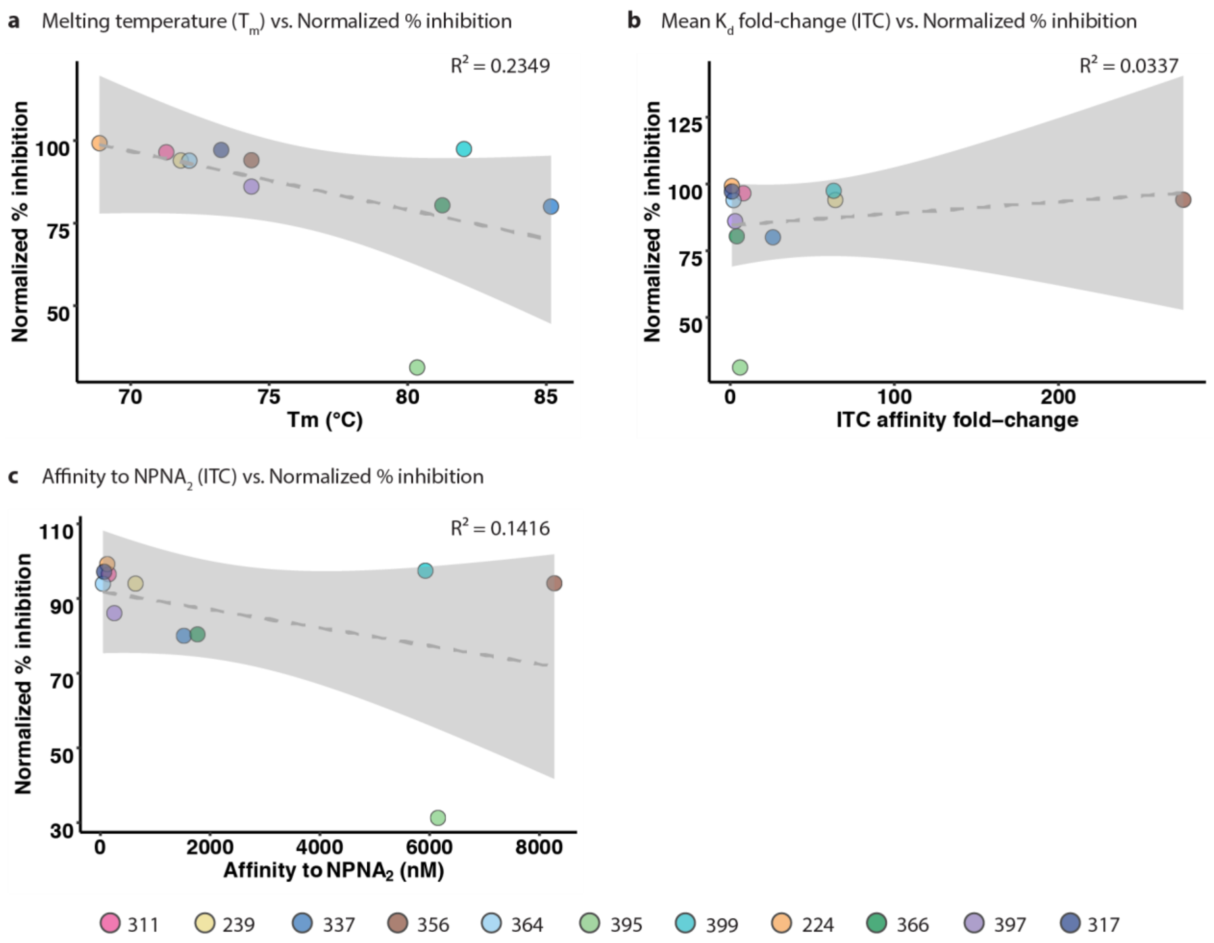

Supplementary Fig. 6. Correlation of other parameters examined in this study with normalized parasite burden. The linear regression graphs plot the % inhibition of the parasite liver burden load, normalized across the two mAb panels, against: **a** Antibody melting temperature (T_m); **b** Mean K_d fold-change from ITC experiments (see also Table 1), and **c** Affinity against the $NPNA_2$ peptide from ITC. The dashed line indicates the fitted linear regression model with 95% confidence interval shaded in grey. Data points for each mAb are colored as shown. Source data are provided as a Source Data file.

Supplementary Table 1. The *p*-values from pairwise comparison of mAbs in the liver burden assay (n = 5 mice) using the two-sided Mann-Whitney U test ($*p = 0.0159$ *or* $0.0318 < 0.05$**) and** $**p = 0.0079 < 0.01$**).**

Panel 1	Naïve	311	239	337	356	364	395
**Naïve**	-						
**311**	0.0079**	-					
**239**	0.0079**	0.0556	-				
**337**	0.0079**	0.0079**	0.0079**	-			
**356**	0.0079**	0.1508	0.8413	0.0079**	-		
**364**	0.0079**	0.1508	0.8413	0.0079**	1.0000	-	
**395**	0.0159*	0.0079**	0.0079**	0.0079**	0.0079**	0.0079**	-

Panel 2	Naïve	311	399	224	366	397	317
**Naïve**	-						
**311**	0.0079**	-					
**399**	0.0079**	0.5476	-				
**224**	0.0079**	0.4206	0.0556	-			
**366**	0.0079**	0.0952	0.0318*	0.0079**	-		
**397**	0.0079**	0.0952	0.0079**	0.0079**	0.6905	-	
**317**	0.0079**	0.6905	0.6905	0.1508	0.0556	0.0079**	-

Supplementary Table 2. Additional isothermal titration calorimetry measurements for anti-NANP antibodies analyzed in this study.

mAb	NPNA$_2$					NPNA$_4$					NPNA$_6$				
	No. of sites	ΔH (cal/mol)	ΔS (cal/ mol·K)	-TΔS (cal/mol)	-ΔG (cal/ mol)	No. of sites	ΔH (cal/mol)	ΔS (cal/ mol·K)	-TΔS (cal/mol)	-ΔG (cal/ mol)	No. of sites	ΔH (cal/mol)	ΔS (cal/ mol·K)	-TΔS (cal/mol)	-ΔG (cal/ mol)
**Fab239**	1.34 ± 0.01	-17963 ± 23	-31.9 ± 0.2	9511 ± 52	-8452 ± 57	0.572 ± 0.011	-46133 ± 437	-120 ± 1.5	35679 ± 455	-10454 ± 631	0.348 ± 0.005	-74853 ± 179	-214 ± 0.6	63903 ± 172	-10950 ± 248
**Fab311**	1.27 ± 0.06	-17990 ± 62	-29.1 ± 0.3	8686 ± 96	-9304 ± 114	0.578 ± 0.002	-43303 ± 155	-109 ± 1.2	32598 ± 144	-10705 ± 212	0.386 ± 0.005	-65990 ± 184	-186 ± 1.0	55456 ± 298	-10534 ± 350
**Fab337**	1.32 ± 0.02	-16670 ± 87	-29.3 ± 0.3	8726 ± 86	-7944 ± 122	0.663 ± 0.006	-37577 ± 5096	-92 ± 15.3	27519 ± 4571	-10058 ± 6846	0.480 ± 0.007	-46407 ± 1529	-122 ± 5.5	36474 ± 1642	-9933 ± 2244
**Fab356**	1.29 ± 0.08	-13543 ± 784	-22.1± 2.5	6599 ± 757	-6944 ± 1090	0.542 ± 0.006	-38697 ± 492	-97 ± 1.7	29020 ± 494	-9677 ± 697	0.354 ± 0.001	-58750 ± 95	-163 ± 0.6	48499 ± 172	-10251 ± 196
**Fab364**	1.41± 0.00	-13877 ± 81	-13.0 ± 0.3	3886 ± 75	-9991 ± 110	0.634 ± 0.008	-36087 ± 281	-86 ± 0.9	25780 ± 277	-10307 ± 395	0.445 ± 0.001	-51720 ± 349	-139 ± 1.2	41343 ± 344	-10377 ± 490
**Fab395**	1.35 ± 0.02	-14510 ± 403	-24.8 ± 1.3	7404 ± 394	-7106 ± 564	0.608 ± 0.032	-36090 ± 3353	-94 ± 11.9	28106 ± 3551	-7984 ± 4884	0.432 ± 0.016	-47443 ± 4643	-132 ± 16.2	39256 ± 4820	-8187 ± 6693
**Fab224**	1.59 ± 0.17	-4811 ± 1147	6.1 ± 4.5	-1819 ± 1343	-6629 ± 197	1.26 ± 0.03	-17267 ± 110	-26.4 ± 0.6	7861 ± 180	-9405 ± 70	0.749 ± 0.003	-31000 ± 122	-72.6 ± 0.5	21646 ± 155	-9354 ± 35
**Fab366**	1.21 ± 0.03	-14447 ± 605	-21.6 ± 2.2	6430 ± 657	-8017 ± 53	1.21 ± 0.01	-17413 ± 176	-32.1 ± 0.7	9561 ± 199	-7853 ± 24	0.670 ± 0.005	-33137 ± 231	-82.2 ± 0.9	24508 ± 255	-8629 ± 24
**Fab397**	1.18 ± 0.04	-14883 ± 579	-24.4 ± 2.1	7285 ± 613	-7599 ± 34	1.3 ± 0.02	-18963 ± 131	-33.4 ± 0.4	9968 ± 120	-8995 ± 31	0.730 ± 0.020	-34947 ± 888	-85.0 ± 3.1	25343 ± 930	-9604 ± 50
**Fab399**	3.23 ± 0.32	-1604 ± 206	16.9 ± 1.0	-5039 ± 295	-6642 ± 89	1.74 ± 0.08	-8052 ± 667	-3.1 ± 2.6	915 ± 772	-7138 ± 107	0.737 ± 0.050	-14867 ± 582	-17.6 ± 2.4	5257 ± 716	-9609 ± 187
**Fab317**	1.36 ± 0.09	−14257 ± 1925	−16.1 ± 7.6	4809 ± 2264	-9448 ± 339	0.95 ± 0.01	−19530 ± 255	−32.8 ± 1.2	9769 ± 359	-9761 ± 106	0.723 ± 0.042	−24417 ± 1263	−50.0 ± 4.2	14917 ± 1251	-9499 ± 122

Supplementary Table 3. X-ray data collection and refinement statistics.

**Data collection**	**Fab399-NPNA$_6$**	**Fab239-NPNA$_4$**	**Fab366-NPNA$_3$**	**Fab399-NPNA$_3$**
Beamline	APS23-IDB	SSRL12-2	APS23-IDD	APS23-IDD
Wavelength (Å)	1.03316	0.97946	1.03320	1.03324
Space group	$P2_1$	$P2_1$	$P2_12_12_1$	$P2_1$
Unit cell parameters (Å, °)	a=62.97, b=85.90, c=89.46	a=82.16, b=55.70, c=115.73	a=59.66, b=68.71, c=108.79	a=63.40, b=87.60, c=89.65
	α=90, β=100.3, γ=90	α=90, β=98.9, γ=90	α=β=γ=90	α=90, β=101.0, γ=90
Resolution (Å)	50.00-2.10 (2.14-2.10)[a]	50.00-2.54 (2.58-2.54)[a]	50.00-1.60 (1.63-1.60)[a]	50.00-1.85 (1.88-1.85)[a]
Unique Reflections	49,542 (1,393)[a]	31,852 (1,613)[a]	56,859 (1,420)[a]	80,717 (3,088)[a]
Redundancy	2.4 (1.8)[a]	3.7 (3.6)[a]	11.7 (2.9)[a]	3.0 (1.9)[a]
Completeness (%)	89.1 (51.0)[a]	91.9 (93.6)[a]	95.1 (48.4)[a]	97.2 (74.4)[a]
$<I/\sigma_I>$	10.3 (1.2)[a]	7.9 (1.6)[a]	28.9 (1.0)[a]	18.6 (1.1)[a]
R_{sym}[b] (%)	8.2 (40.7)[a]	14.5 (89.8)[a]	7.6 (55.9)[a]	7.9 (48.4)[a]
R_{pim}[b] (%)	6.0 (33.3)[a]	8.2 (52.2)[a]	2.2 (32.6)[a]	5.2 (38.0)[a]
$CC_{1/2}$[c] (%)	90.1 (71.5)[a]	88.0 (52.1)[a]	95.4 (71.9)[a]	91.7 (69.2)[a]
**Refinement statistics**				
Resolution (Å)	44.01-2.10	32.81-2.54	45.05-1.60	46.75-1.85
Reflections (work)	49,513	31,820	56,784	80,521
Reflections (test)	2,434	1,591	2,793	3,869
R_{cryst}[d] / R_{free}[e] (%)	19.6/24.6	19.5/24.6	18.9/21.1	18.6/22.5
**No. of atoms**				
Fab	6,502	6,475	3,325	6,645
Peptide	168	115	79	158
Water	347	106	265	367
**Average B-value (Å^2)**				
Fab	37	42	24	39
Peptide	44	36	31	41
Water	38	36	33	42
Wilson B-value	32	36	22	29
**RMSD from ideal geometry**				
Bond length (Å)	0.005	0.002	0.007	0.007
Bond angle (°)	0.73	0.58	0.90	0.85
**Ramachandran statistics**[f]				
Favored (%)	98.17	97.34	97.73	98.17
Outliers (%)	0.11	0.00	0.00	0.11

[a] Numbers in parentheses refer to the highest resolution shell.
[b] $R_{sym} = \Sigma_{hkl} \Sigma_i | I_{hkl,i} - <I_{hkl}> | / \Sigma_{hkl} \Sigma_i I_{hkl,i}$ and $R_{pim} = \Sigma_{hkl} (1/(n-1))^{1/2} \Sigma_i | I_{hkl,i} - <I_{hkl}> | / \Sigma_{hkl} \Sigma_i I_{hkl,i}$, where $I_{hkl,i}$ is the scaled intensity of the ith measurement of reflection h, k, l, $<I_{hkl}>$ is the average intensity for that reflection, and *n* is the redundancy.
[c] $CC_{1/2}$ = Pearson correlation coefficient between two random half datasets.
[d] $R_{cryst} = \Sigma_{hkl} | F_o - F_c | / \Sigma_{hkl} | F_o | \times 100$, where F_o and F_c are the observed and calculated structure factors, respectively.
[e] R_{free} was calculated as for R_{cryst}, but on a test set comprising 5% of the data excluded from refinement.
[f] From MolProbity[1].

Supplementary Table 4. X-ray data collection and refinement statistics (continued).

Data collection	Fab239-NPNA$_2$	Fab356-NPNA$_2$	Fab364-NPNA$_2$	Fab395-NPNA$_2$	Fab224-NPNA$_4$
Beamline	APS23-IDD	APS23-IDD	APS23-IDD	APS23-IDD	APS23-IDD
Wavelength (Å)	1.03321	1.03321	1.03320	1.03324	1.03324
Space group	$P4_32_12$	$P2_12_12_1$	$C222_1$	$P2_12_12_1$	$P2_1$
Unit cell parameters (Å, °)	a= b=121.52, c=82.61	a=64.14, b=81.76, c=84.05	a=80.44, b=117.05, c=116.91	a=60.42, b=78.66, c=90.67	a=42.92, b=67.34, c=84.77
	α= β=γ=90	α=β= γ=90	α=β=γ=90	α=β=γ=90	α=90, β= 96.1, γ=90
Resolution (Å)	50.00-1.85 (1.88-1.85)[a]	50.00-2.52 (2.56-2.52)[a]	50.00-2.10 (2.14-2.10)[a]	50.00-2.60 (2.64-2.60)[a]	50.00-1.23 (1.25-1.23)[a]
Unique Reflections	52,998 (2,623)[a]	15,560 (773)[a]	28,464 (556)[a]	13,944 (675)[a]	114,397 (855)[a]
Redundancy	13.3 (9.8)[a]	7.0 (6.2)[a]	5.3 (1.8)[a]	6.7 (6.9)[a]	2.9 (1.0)[a]
Completeness (%)	100.0 (100.0)[a]	100.0 (100.0)[a]	86.3 (34.8)[a]	99.9 (100.0)[a]	81.7 (12.4)[a]
$\langle I/\sigma_I \rangle$	18.6 (1.9)[a]	13.6 (1.6)[a]	13.3 (2.0)[a]	8.0 (2.0)[a]	19.1 (1.4)[a]
R_{sym}[b] (%)	18.8 (93.3)[a]	14.4 (105.3)[a]	10.8 (40.0)[a]	21.6 (124.4)[a]	5.4 (35.7)[a]
R_{pim}[b] (%)	5.3 (30.3)[a]	5.8 (45.5)[a]	4.8 (31.3)[a]	9.0 (52.3)[a]	3.4 (35.3)[a]
$CC_{1/2}$[c] (%)	95.1 (72.5)[a]	90.0 (54.7)[a]	93.4 (73.8)[a]	88.3 (49.6)[a]	89.3 (67.4)[a]
**Refinement statistics**					
Resolution (Å)	48.97-1.85	43.27-2.52	43.85-2.10	47.92-2.60	42.68-1.23
Reflections (work)	52,931	15,512	28,419	13,902	114,353
Reflections (test)	2577	748	1,431	699	5,705
R_{cryst}[d] / R_{free}[e] (%)	18.7/20.6	20.9/25.6	21.3/25.6	19.8/24.4	16.9/18.3
**No. of atoms**					
Fab	6,499	3,281	3,135	3,202	3,355
Peptide	107	56	56	43	84
Protein G	882	N/A	453	N/A	N/A
Water	349	22	123	60	491
**Average B-value (Å^2)**					
Fab	24	56	53	41	18
Peptide	17	75	78	45	17
Protein G	26	N/A	45	N/A	N/A
Water	28	46	42	30	30
Wilson B-value	20	48	37	37	11
**RMSD from ideal geometry**					
Bond length (Å)	0.009	0.004	0.004	0.002	0.006
Bond angle (°)	0.87	0.66	0.67	0.60	0.85
**Ramachandran statistics**[f]					
Favored (%)	97.79	96.58	95.18	95.83	97.77
Outliers (%)	0.00	0.00	0.21	0.23	0.00

[a] Numbers in parentheses refer to the highest resolution shell.
[b] $R_{sym} = \Sigma_{hkl} \Sigma_i | I_{hkl,i} - \langle I_{hkl} \rangle | / \Sigma_{hkl} \Sigma_i I_{hkl,i}$ and $R_{pim} = \Sigma_{hkl} (1/(n-1))^{1/2} \Sigma_i | I_{hkl,i} - \langle I_{hkl} \rangle | / \Sigma_{hkl} \Sigma_i I_{hkl,i}$, where $I_{hkl,i}$ is the scaled intensity of the ith measurement of reflection h, k, l, $\langle I_{hkl} \rangle$ is the average intensity for that reflection, and *n* is the redundancy.
[c] $CC_{1/2}$ = Pearson correlation coefficient between two random half datasets.
[d] $R_{cryst} = \Sigma_{hkl} | F_o - F_c | / \Sigma_{hkl} | F_o |$ x 100, where F_o and F_c are the observed and calculated structure factors, respectively.
[e] R_{free} was calculated as for R_{cryst}, but on a test set comprising 5% of the data excluded from refinement.
[f] From MolProbity[1].

Supplementary Table 5. Germline genes and buried surface area (BSA) of mAbs analyzed in this study.

mAb	HC *V* gene (IGVH)	HC *D* gene	HC *J* gene	LC *V* gene (IGKV, IGLV)	LC *J* gene	HC BSA (Å^2)	LC BSA (Å^2)	Total BSA (Å^2)
239	*3-33*03 or*04*	*D2-21*02*	*J5*01*	*KV1-5*01 or KV1-5*03*	*KJ2*02 or KJ5*01*	386	164	550
311	*3-33*01 or *06*	*D3-22*01*	*J4*02 or J4*03*	*LV1-40*01 or LV1-40*02*	*LJ3*02*	366	107	473
337	*3-33*03, 3-30*04,*14 or *16 3-30-3*03*	*D2-21*01*	*J3*02*	*KV3-15*01*	*KJ1*01*	N/A	N/A	N/A
356	*3-33*03*	*D3-22*01*	*J4*02 or J4*03*	*KV3-15*01*	*KJ3*01*	457	115	572
364	*3-33*03, 3-30*02, or 3-30-5*02*	*D4-17*01*	*J4*02*	*KV1-5*03*	*KJ1*01*	344	99	443
395	*3-33*03*	*D3-9*01*	*J4*02 or J4*03*	*KV1-5*01*	*KJ1*01*	379	41	420
224	*3-49*04*	*D4-17*01*	*J6*01*	*LV1-40*01*	*LJ3*02*	429	188	617
399	*3-49*03*	-	*J2*01*	*KV2-29*02 or KV2D-29*02*	*KJ1*01*	314	178	492
366	*1-2*02*	*D2-21*01*	*J3*02*	*KV1-27*01*	*KJ1*01*	348	228	576
317 (*VH3-30*)	*3-30*14 or *16*	*D3-16*01*	*IJ4*02*	*KV1-5*01*	*KJ1*01*	310	209	519
397 (*VH3-15*)	*3-15*01*	*D3-3*01*	*J4*02 or J4*03*	*KV2-28*01 or KV2D-28*01*	*KJ1*01, KJ2*01 or KJ5*01*	264	329	593

Supplementary Table 6. Conserved positions and corresponding residues of *IGHV3-33* mAbs that interact with NPNA peptide

mAb	227	239	311	337†	356	364	395	Germline residue	Interaction
H 31 (32)‡	A	N	N	T	N*	G	C	S	Conserved MHB
H 32 (33)‡	F	F	Y	Y	F	Y	Y	Y	Conserved vdW
H 33 (34)‡	G	G	G	G	G	G	G	G	Conserved MHB
H 50	V	V	**I**	L	V	**I**	V	V	**Evolved vdW**
H 52	W	W	W	W	W	W	W	W	Conserved CH-pi
H 52A	Y	H	Y	H	H	F	H (also SHB)	Y	Conserved MHB
H 58	Y	F	F	F	F	Y	H	Y	Conserved vdW
H 95	V	D (SHB)	A (MHB)	D	D (SHB)	V	A (MHB)	-	Varied as indicated

*The main chain of $^{H}Asn^{31}$ in mAb356 does not form a hydrogen bond with the peptide.
†Residues in 337 are included for complete comparison but are in grey as no structure of this mAb is available.
‡Residue number for mAb395.
MHB = Hydrogen bond between the Fab main chain and NANP peptide.
SHB = Hydrogen bond between the Fab side chain and NANP peptide.
vdW = van der Waals interaction.

Supplementary reference

1 Chen, V. B. *et al.* MolProbity: all-atom structure validation for macromolecular crystallography. *Acta Crystallogr D Biol Crystallogr* **66**, 12-21 (2010).
