# Supplementary material for: Structural and biophysical correlation of anti-NANP antibodies with in vivo protection against P. falciparum
Source: Nat Commun. 2021 Feb 16;12:1063. doi: 10.1038/s41467-021-21221-4 (PMC7887213; doi:10.1038/s41467-021-21221-4)
Supplement: Supplementary file 2 — Reporting Summary [file 41467_2021_21221_MOESM2_ESM.pdf]

nature research

Corresponding author(s): Ian A. Wilson

Last updated by author(s): Dec 22, 2020

Reporting Summary

Nature Research wishes to improve the reproducibility of the work that we publish. This form provides structure for consistency and transparency in reporting. For further information on Nature Research policies, see our Editorial Policies and the Editorial Policy Checklist.

Statistics

For all statistical analyses, confirm that the following items are present in the figure legend, table legend, main text, or Methods section.

n/a	Confirmed	
☐	☒	The exact sample size (n) for each experimental group/condition, given as a discrete number and unit of measurement
☐	☒	A statement on whether measurements were taken from distinct samples or whether the same sample was measured repeatedly
☒	☐	The statistical test(s) used AND whether they are one- or two-sided<br>*Only common tests should be described solely by name; describe more complex techniques in the Methods section.*
☒	☐	A description of all covariates tested
☒	☐	A description of any assumptions or corrections, such as tests of normality and adjustment for multiple comparisons
☐	☒	A full description of the statistical parameters including central tendency (e.g. means) or other basic estimates (e.g. regression coefficient) AND variation (e.g. standard deviation) or associated estimates of uncertainty (e.g. confidence intervals)
☐	☒	For null hypothesis testing, the test statistic (e.g. F, t, r) with confidence intervals, effect sizes, degrees of freedom and P value noted<br>*Give P values as exact values whenever suitable.*
☒	☐	For Bayesian analysis, information on the choice of priors and Markov chain Monte Carlo settings
☒	☐	For hierarchical and complex designs, identification of the appropriate level for tests and full reporting of outcomes
☒	☐	Estimates of effect sizes (e.g. Cohen's d, Pearson's r), indicating how they were calculated

Our web collection on statistics for biologists contains articles on many of the points above.

Software and code

Policy information about availability of computer code

Data collection	For crystal structure determination, the datasets were indexed, integrated, and scaled with HKL2000 package. The structures were determined by molecular replacement using Phaser. Structure refinement was performed using phenix.refine and iterations of refinement using Coot.
Data analysis	Data analysis in Figures 1 and 3 was performed in R version 4.0.0 with open source packages specified in the statistical analysis section of the paper. The isothermal titration calorimetry and differential scanning calorimetry data were analyzed using Origin 7.0 software, whereas the bio-layer interferometry data were analyzed using Octet Red Data Analysis software version 9.0.

For manuscripts utilizing custom algorithms or software that are central to the research but not yet described in published literature, software must be made available to editors and reviewers. We strongly encourage code deposition in a community repository (e.g. GitHub). See the Nature Research guidelines for submitting code & software for further information.

Data

Policy information about availability of data

All manuscripts must include a data availability statement. This statement should provide the following information, where applicable:

- Accession codes, unique identifiers, or web links for publicly available datasets
- A list of figures that have associated raw data
- A description of any restrictions on data availability

The crystal structures of all Fab-peptide complexes have been deposited in the Protein Data Bank with accession codes: 6W00 (Fab239-NPNA2), 6W05 (Fab356-NPNA2), 6WFW (Fab364-NPNA2), 6WFX (Fab395-NPNA2), 6WFY (Fab224-NPNA4), 6WFZ (Fab399-NPNA3), 6WG0 (Fab366-NPNA3), 6WG1 (Fab399-NPNA6), and 6WG2 (Fab239-NPNA4). These raw data are associated with Figures 2, and 4-7. Crystal structures of Fab1210, MGG4, CIS43, 1450, and 317 and 397 used in Figures 6-7 were obtained from previous studies (PDB ID: 6D01, 6BQB, 6B5O, 6D11, 6AXL, and 6UC5, respectively). Cryo-EM structures of Fab311 in complex with rsCSP

used in Figure 6 was also obtained from the previous study (PDB ID: 6MB3).

Field-specific reporting

Please select the one below that is the best fit for your research. If you are not sure, read the appropriate sections before making your selection.

☒ Life sciences ☐ Behavioural & social sciences ☐ Ecological, evolutionary & environmental sciences

For a reference copy of the document with all sections, see nature.com/documents/nr-reporting-summary-flat.pdf

Life sciences study design

All studies must disclose on these points even when the disclosure is negative.

Sample size	Based on published studies (DOI: 10.1186/s12936-019-3055-9 and DOI: 10.1186/s12936-020-03181-0), we have determined that the sample size that we use allow us to define differences between groups.
Data exclusions	No data were excluded from the analysis
Replication	Most of the antibodies were evaluated with the two in vivo assays more than once and showed consistent outcomes with the data reported in Figure 1. In particular, the assessment of in vivo protection of mAb311, 317 and 397 was also previously reported in our published studies (DOI: 10.1073/pnas.1715812114 and DOI: 10.1016/j.jmb.2019.12.029). Isothermal titration calorimetry was performed in triplicate, all of which display consistent results. Structure determination, bio-layer interferometry, and differential scanning calorimetry were not replicated.
Randomization	For protection study experiments, all groups of mice were matched by age and sex.
Blinding	In the experiments conducted to measure parasite load and protection from infection in mice, the antibodies were blinded. The investigators doing the mice experiments received the antibodies from us coded and we decoded only after the results were obtained back from our co-authors. Blinding is not relevant to the structural and biophysical experiments as the identity of the antibody needs to be known to properly assess its structure, affinity, and thermal stability.

Reporting for specific materials, systems and methods

We require information from authors about some types of materials, experimental systems and methods used in many studies. Here, indicate whether each material, system or method listed is relevant to your study. If you are not sure if a list item applies to your research, read the appropriate section before selecting a response.

Materials & experimental systems

n/a	Involved in the study	
☐	☒	Antibodies
☐	☒	Eukaryotic cell lines
☒	☐	Palaeontology and archaeology
☐	☒	Animals and other organisms
☒	☐	Human research participants
☐	☒	Clinical data
☒	☐	Dual use research of concern

Methods

n/a	Involved in the study	
☒	☐	ChIP-seq
☒	☐	Flow cytometry
☒	☐	MRI-based neuroimaging

Antibodies

Antibodies used

anti-CD3-FITC (BioLegend, cat# 300406, clone UCHT1)
anti-CD14-FITC (BioLegend, cat# 325604, clone HCD14)
anti-CD19 -BV421 (BioLegend, cat# 302234, clone HIB19)
anti-CD20-PerCP/cy5.5 (BD, cat# 340955, clone L27)
anti-CD27-BV510 (BioLegend, cat# 302836, clone O323)
anti-CD38-PE/cy7 (BioLegend, cat# 356607, clone HB-7)
anti-IgA-FITC (Miltenyi, cat# 130-113-475, clone IS11-8E10)
anti-IgM-APC/cy7 (BioLegend, cat# 314520, clone MHM-88)
anti-CD3-FITC (BioLegend, cat# 300406, clone UCHT1)
anti-CD14-FITC (BioLegend, cat# 325604, clone HCD14)
anti-CD19 -BV421 (BioLegend, cat# 302234, clone HIB19)
anti-CD20-PerCP/cy5.5 (BD, cat# 340955, clone L27)
anti-CD27-BV510 (BioLegend, cat# 302836, clone O323)
anti-CD38-PE/cy7 (BioLegend, cat# 356607, clone HB-7)
anti-IgA-FITC (Miltenyi, cat# 130-113-475, clone IS11-8E10)

	anti-IgM-APC/cy7 (BioLegend, cat# 314520, clone MHM-88)
Validation	We did not validate commercial antibodies and relied on the target validation stated by the manufacturer. The information is accessible on the manufacturers' websites under Cat#.

Eukaryotic cell lines

Policy information about cell lines

Cell line source(s)	ExpiCHO cells are from ThermoFisher-Life Technologies (catalog number: A29127).
Authentication	We did not authenticate the commercial cell line.
Mycoplasma contamination	The cell line used was not tested for mycoplasma contamination.
Commonly misidentified lines (See ICLAC register)	No commonly misidentified cell lines were used in the study.

Animals and other organisms

Policy information about studies involving animals; ARRIVE guidelines recommended for reporting animal research

Laboratory animals	Studies using mice were carried out using 6-8 weeks old C57BL/6 females, maintained at the animal facility of the Johns Hopkins Bloomberg School of Public Health. Mice rooms are kept at 40-60% relative humidity at a temperature of 68-79ºF with at least 10 room air changes per hour. Mice have a cycle of 14 hours light and 10 hours darkness.
Wild animals	No wild animals were used in the study.
Field-collected samples	No field-collected samples were used in the study.
Ethics oversight	The assays using mice were performed in strict accordance with the recommendations in the Guide for the Care and Use of Laboratory Animals of the National Institutes of Health. The protocol was approved by the Animal Care and Use Committee of the Johns Hopkins University, protocol number MO18H419.

Note that full information on the approval of the study protocol must also be provided in the manuscript.

Clinical data

Policy information about clinical studies

All manuscripts should comply with the ICMJEguidelines for publication of clinical research and a completedCONSORT checklist must be included with all submissions.

Clinical trial registration	Clinical Trials.gov identifier: NCT01857869
Study protocol	The study protocol can be accessed on https://www.clinicalstudydatarequest.com/ with identifier: 117014. Please refer to the GSK Clinical Study Register.
Data collection	Location : United States, Maryland, Silver Spring, 20910<br>Actual Study Start Date : May 20, 2013<br>Actual Primary Completion Date : March 24, 2014<br>Actual Study Completion Date : December 16, 2014
Outcomes	Clinical outcomes are reported at clintrials.gov with the identifier provided above. The outcomes are also reported in the following publication:<br>Regules, J. A. et al. Fractional third and fourth dose of RTS,S/AS01 malaria candidate vaccine: a phase 2a controlled human malaria parasite infection and immunogenicity study. J Infect Dis 214, 762-771 (2016).
